# Investigating Development in Human Evolution: Specificities, Challenges, and Opportunities

**DOI:** 10.1002/evan.70001

**Published:** 2025-03-03

**Authors:** Mathilde Lequin, Thomas Colard, Antony Colombo, Adeline Le Cabec, Floriane Remy, Alexandra Schuh

**Affiliations:** ^1^ Univ. Bordeaux, CNRS, MCC PACEA, UMR 5199 Pessac France; ^2^ Univ. Lille, CHU Lille Department of Oral and Maxillofacial Radiology Lille France; ^3^ Univ. Bordeaux Montaigne, CNRS, Univ. Bordeaux Archéosciences Bordeaux, UMR 6034 Pessac France; ^4^ EPHE PSL University Paris France; ^5^ Department of Human Origins Max Planck Institute for Evolutionary Anthropology Leipzig Germany

**Keywords:** developmental biology, developmental plasticity, Extended Evolutionary Synthesis, growth, life history, maturation, ontogeny, paleoanthropology

## Abstract

Unlike developmental biologists, paleoanthropologists primarily investigate development using skeletal remains, specifically fossilized and already‐formed bones and teeth. Focusing on peri‐ and/or postnatal growth, they reconstruct development from fragmented “snapshots” of individual trajectories at various ontogenetic stages. These constraints prompt a discussion of what defines development versus growth, and its boundaries in studies of hominin evolution. We explore how paleoanthropologists address the limitations of the fossil record by using diverse methodological and theoretical frameworks to identify developmental markers despite missing data. Finally, we discuss the potential of the “Extended Evolutionary Synthesis,” which calls for a greater focus on developmental processes in interpreting phenotypic variation in the fossil record.

## 1 Introduction

Paleoanthropological studies of hominin development are crucial for understanding what defines our species *Homo sapiens*, as evolutionary changes between species may arise directly or indirectly from developmental processes. Development in paleoanthropology can only be defined on the basis of what can be accessed through the fossil remains, i.e. only certain periods of ontogenesis, and only mineralized tissues. A significant emphasis has been placed on the opportunities provided by a growing fossil record of subadults to identify the developmental patterns shared among or unique within hominin taxa [1]. Advances in developmental biology, from the genetic mechanisms of development to the epigenetic effects of environmental inputs, have also sparked a growing interest among paleoanthropologists, working to integrate these insights into their understanding of anatomical variations in hominins [2, 3, 4, 5]. Our approach in this paper does not apply developmental biology frameworks to the study of human evolution by analogy, but rather starts from paleoanthropologists’ practices to explore how their questions about development align with some of those in developmental biology.

Working with scarce and often fragmented fossil remains, paleoanthropologists rely solely on morphological data from bones and teeth, which are available only during late prenatal and postnatal ontogeny. As a result, their understanding of development primarily focuses on the growth and maturation of the late fetus into adulthood. By contrast, developmental biologists rely mostly on molecular (i.e. genetic, hormonal) and cellular data, which are available from the earliest stages of life and in extensive developmental series [6]. In developmental biology, “development” mainly refers to the formation of the embryo and the suite of processes leading to a fetus and newborn [6].

This paper examines how the specificities of the fossil record shape paleoanthropologists’ understanding of development and contribute, alongside developmental biology, to our broader understanding of ontogenetic processes and patterns. The first part of this review explores the limitations of the fossil record, showing how they prompt us to rethink a framework for studying development that must be inferred from fossilized remains. The second part investigates the diverse strategies, based on various methodological and theoretical frameworks, used by paleoanthropologists to overcome the inherent limitations of the fossil record and to extract maximum evidence from limited data. Finally, we address the current challenges of further integrating these new insights into the interpretation of hominin evolution, in the context of contemporary biological discussions on the developmental origins of phenotypic variations and morphological innovations.

## 2 Developmental Studies in Past Populations: Little Data, Many Challenges

### 2.1 Defining Development in Paleoanthropology

In developmental biology, “development” usually refers to the sequence of changes going from fertilization to adulthood, with a focus on the earliest stages in the formation of the embryo, fetus, and newborn. However, biologists and philosophers have raised the question of the temporal and spatial boundaries of development [7, 8]. Although the traditional view holds that development stops once the organism reaches adulthood, seen as the realization of the egg's internal potentialities, it has been suggested that development continues throughout life and is strongly influenced by the environment [7]. Consider, for instance, an organism's ability to change in response to environmental conditions—i.e., phenotypic plasticity (see Box 1)—whether due to prenatal stress or postnatal stimuli.

Box 1 **The example of body height: The result of intertwined multifactorial processes**

Under favorable nutritional conditions, height appears to be primarily genetically regulated [9]. At birth, 30% of adult height is attained, while less than 5% of the total growth time has elapsed. The pubertal period, although longer, contributes only about 20% to adult height [10]. Population variation in height is attributed to a combination of genetic and environmental factors. Although the maximum height an individual can reach is genetically determined [11], considerable variability is observed. Differences are noted between the adult height of men and women, primarily due to a shorter childhood period in girls. As puberty begins earlier in girls (9 to 15 years) than in boys (11 to 18 years), growth also ceases earlier [12].

Groups from different geographical origins, within similar socioeconomic and environmental contexts, exhibit equivalent height averages. Before adolescence, growth is more sensitive to the environment. Children from populations with different genetic backgrounds but living in similar environments tend to have the same height. During adolescence, genetic determinants of growth are more pronounced, and differences between populations become visible in the same environment. There is less variation in height for the same socioeconomic, dietary, and environmental contexts during childhood than during an individual's adolescence, again implicating genetic factors [13, 14]. Human height is influenced by both genetic and environmental factors, which are closely interconnected and interdependent, illustrating the plasticity of growth, as well as underscoring the many variables to be considered when interpreting fossil hominin skeletal remains.

In paleoanthropology, “development” refers to the sequence of changes in late prenatal and postnatal ontogeny, affecting the shape and size of bones and teeth, both in external and internal structure [15]. Development is thus understood as the result of both growth and maturation. Growth is a quantitative process from subadult to adult size. Conversely, maturation is a qualitative process towards the mature state, typically assessed through the skeletal modifications resulting from hormone levels and pubertal signs [12, 16]. Development is also sometimes more narrowly defined as changes in shape, and growth as changes in size over time [17].

In this context, teeth are a treasure trove of information on hominin development. Tooth development involves the incremental mineralization of enamel and dentine, followed by maturation [18, 19]. Each tooth begins forming at a specific age and in a specific order. In primates, including humans, deciduous teeth begin forming *in utero* and continue developing after birth, typically erupting by 3 years of age in modern humans. They then start resorbing their roots as the forming permanent teeth erupt from within the alveolar bone into the oral cavity. One may assume that tooth development is complete in early adulthood [19]. However, cementum layers are laid down on tooth roots roughly at the time of tooth emergence until the death of the individual [20]. Could this process be considered a facet of development? The study of cementum, which forms incrementally until death, provides information on the chronological age of the individual (cementochronology [21]). Because cementum provides insights into adult life history, this tooth tissue could be seen as extending development beyond adult maturity into later stages of life.

Bone is another mineralized tissue that provides valuable insights into development (see Box 2), although bone tissues develop following different mechanisms than dental tissues. The process of bone remodeling has important implications in the natural renewal of the bones as well as in bone repair and homeostasis [22]. It is composed of two simultaneous and interdependent mechanisms: bone deposition (or formation), with the activity of osteoblasts; and bone resorption, with the activity of osteoclasts [30]. Thus, paleoanthropologists understand development as changes in mineralized tissues throughout postnatal life.

Box 2 **Bone plasticity and the boundaries of development**

After maturity, skeletal growth slows down significantly and stops. However, bone continues to remodel throughout life due to genetic, hormonal, and environmental factors [22]. Mechanical loadings can affect the activities of bone deposition and resorption, leading to changes in shape and internal structures [23]. Studies of bone functional adaptation explore how bones adjust to mechanical demands. This approach highlights the limits of the definition of development, usually defined as beginning at fertilization and ending at adulthood. By producing phenotypic variability, functional adaptation introduces plasticity and requires examining how the environment influences morphology. This brings attention to ongoing debates on differing developmental perspectives in paleoanthropology: on one hand, it is viewed that morphological variation is mostly genetically predetermined. This suggests that bone morphology is shaped by the positional information given to cartilaginous cells. Under this view, functional adaptation is considered to only play a minor role in development [2].

Conversely, studies on bone functional adaptation, particularly those investigating limb internal structures and cross‐sectional geometry, offer examples of how environmental factors can play a role in the phenotypic plasticity [24]. While this process inherently involves changes in gene expression that influence skeletal traits, studies on bone functional adaptation primarily focus on how morphology is shaped by biomechanics and physical activities (e.g., locomotor behaviors, tool use). In particular, the trabecular bone studies have shown how bone structures adapt from the very beginning of development [25] until adulthood [26] under the effect of environmental and cultural factors, although there exists a common pattern in the trabecular bone modeling during early ontogeny [27]. This made it possible to track psychomotor development stages [28] and locomotor behavior changes, bringing important new insights into bone functional adaptation. Thus, researchers can make functional inferences from variations in internal bone microstructure [26], but also investigate the potential influence of these biomechanical responses on development [29].

Exploring the definition of development in paleoanthropology also involves considering the scale at which this process is addressed. Paleoanthropologists are typically not dealing with organisms, but with fragmentary remains of organisms that died long ago. It has been argued that in paleoanthropology, the term “development” is used to characterize species‐level adaptations and features, in contrast to the disciplines studying individual development [17]. The main challenge is that paleoanthropologists rely on the remains of individuals, with intragroup variability that is not fully known, to address the developmental features of the population, species, or genus. The scale varies from the short term, i.e. the life of the individual, to the long term, i.e. the evolution of the taxon, with the challenge of not inferring too readily from the individual to the taxon [31].

### 2.2 Fossil Specimens as Instant Snapshots of Developmental Trajectories

Sciences that study past organisms and their biological processes, like paleoanthropology, often grapple with “epistemic scarcity”—a condition marked by the rarity and fragility of available data [32]. In the case of hominin development, paleoanthropologists face extreme epistemic scarcity, relying almost entirely on dental and skeletal remains to extract as much information as possible about ontogenetic processes. Most of the time, information from soft tissues or under‐mineralized bones (e.g., from prenatal individuals) is lost, leaving no data on the earliest developmental stages. With regard to skeletal data, paleoanthropologists very often grapple with both limited sample sizes and/or damaged partial remains. Indeed, the extent of the fossil record is dependent on the archeological discoveries and taphonomic processes implying various preservation rates. These rates can vary according to the anatomical part considered, but also to the ontogenetic stage (e.g., perinate bones are notably more fragile compared to adult bones). To date, there are very few remains of juvenile early *Homo* available for study (e.g [33]). In contrast, several sub‐complete perinates and infants from Neanderthals have been recovered (e.g., Le Moustier 2, Mezmaiskaya 1 [34]) although these remain exceptional findings. A handful of sites have delivered the osteological remains of numerous individuals belonging to several age classes, among which are the Sima de Los Huesos in Spain [35], or the Dinaledi Chamber and the Lesedi Chamber in South Africa [36]. Although there is ongoing debate about whether these samples represent reliable overviews of natural populations, small sample sizes are nonetheless the most common situation encountered in paleoanthropology (e.g. *A. sediba*, for which only one juvenile and one adult have been described [37]). These limitations in both quantity and quality of the fossil record constrain the statistical tools available, and influence or limit the interpretations that can be drawn about hominin evolutionary history across time and space, at the intra‐ and inter‐individual and specific levels [38]. Mathematical modeling and missing data estimation are increasingly explored to compensate for these sources of bias (e.g [39, 40].; see Section 3.2). Moreover, taxonomic attributions can be difficult, since some diagnostic morphological features may not be as expressed in subadults as in adults [41, 42]. Finally, it is often unclear if fossils represent healthy individuals with a normal development who died from non‐pathological causes. A very famous case is the Taung infant who was likely hunted by a predator, potentially a bird of prey [43]. However, the fossils may represent individuals that died from a disease which did not leave specific marks on their skeleton, but may have affected their development—a situation coined as “the osteological paradox” [44].

Considering these limitations, any longitudinal or semi‐longitudinal approach, such as those conducted on clinical examinations on present‐day humans [45, 46], is obviously not possible in fossils. Fossil remains represent “instant snapshots” of a developmental trajectory [47], which is mostly a hypothetical line drawn from single data points. To address this constraint, a cross‐sectional approach is required. That is, several specimens from the same taxon, deceased at different ontogenetic stages, are grouped into age classes based on predefined criteria, such as dental eruption patterns or calcification stages, skull synchondroses or long bones stages of maturation [48].

However, organizing specimens into age cohorts introduces additional challenges. This approach reduces the sample size within each age group, and potentially aggregates specimens from widely separated times and locations that may not represent a reliable biological population. Potential age group misassignments may occur due to the variation between individuals and the difficulty of distinguishing between different sources of morphological variability in subadult specimens. These issues can affect comparisons of ontogenetic trajectories across different species. Similarly, fossils are often compared to the known variation of extant species, such as present‐day humans and non‐human great apes. As growth can be quite variable from one human population to another, the comparative sample that is selected can lead to differential interpretations on the growth and developmental patterns of fossils. For example, it is still debated whether Neanderthals grew faster than present‐day humans, or whether they fall within the variation known for the latter [49, 50]. This is partly due to the fact that two important biological factors, chronological age and sex, are amongst the most difficult to determine in subadult fossils [41]. This challenge includes that sexual dimorphism may not be fully expressed at that stage. Recent advances in molecular biology, such as paleogenetics and paleoproteomics, can now facilitate sex determination using aDNA and proteins extracted from bone and dental remains when preserved and to a certain time point [51]. For age estimation, the analysis of inner dental microstructures can help calculate an age at death with unprecedented precision (see Section 3.2).

## 3 Strategies Deployed in Paleoanthropology to Overcome the Limitations of the Fossil Record

### 3.1 Combining Theoretical and Methodological Frameworks

Paleoanthropologists’ strategies for investigating developmental patterns exemplify what Currie [52] describes as “methodological omnivory”, meaning the ability to draw from several frameworks to address situations where data is scarce. Here we focus on two research fields that paleoanthropologists are mobilizing to study hominin development, taking into consideration the numerous constraints described in the previous section: evolutionary developmental biology (Evo‐Devo) and auxology.

Evo‐Devo may be defined as the branch of biology “concerned with how and why organismal development matters to evolution” [5]. It revolves around two main themes:

1. The evolution of development, examining how patterns and processes of development have changed over time, and how they drive phenotypic diversification at macroevolutionary scales
2. The developmental basis of evolution, which explores how ontogenetic processes may impact evolutionary trajectories, and how patterns of phenotypic variation in populations may bias or constrain the rate and direction of evolutionary changes within or between species [5, 53].

By shifting the focus from anatomical traits to the developmental mechanisms underlying morphological adaptations, Evo‐Devo is expected to have a significant impact on how paleoanthropologists make functional and phylogenetic inferences from fossil anatomy [2]. The emergence of an “Evolutionary Developmental Paleoanthropology” [4] and “Evolutionary Developmental Anthropology” [5] has helped frame key research questions about the hominin fossil record around two main axes:

1. The evolution of development across hominin taxa: what is the ontogeny of the different hominin taxa? What is the evolutionary history of the different ontogenetic patterns that can be highlighted based on the fossil record?
2. The role of development in the evolution of traits that are shared within hominin taxa: how have developmental mechanisms generated, biased, or constrained the morphological diversity within the hominin clade? What ontogenetic traits are shared across hominin taxa, and which are unique to specific hominin taxa, including *Homo sapiens*?

With this theoretical agenda goes a methodological framework, incorporating specific concepts and tools. Key concepts such as allometry, heterochrony, modularity, and developmental instability (see Box 3) are essential for applying Evo‐Devo to paleoanthropology. These concepts enable comparative studies of developmental patterns in extant primates to be applied to hominins [5], helping to address the limitations of the fossil record. Using homologous landmarks and semilandmarks, geometric morphometrics enables quantifying morphological variation of biological traits, both in terms of size (i.e., dimensions) and conformation (i.e., shape). This approach also allows the visualization of complex shape and ontogenetic processes, and the exploration of how this shape variation correlates with environmental, genetic, and behavioral variables [71, 72, 73].

Box 3 **Glossary**

*Allometry*

Changes in size during ontogeny and throughout evolution are important factors affecting morphological variation. Allometry is the covariation between changes in shape and size [54, 55] and has been widely studied in paleoanthropology. One can distinguish three types of allometry [56, 57]. *Ontogenetic allometry* describes the covariation of features across ontogenetic stages within a species. *Static allometry* relates to the covariation of features within a single ontogenetic stage. Finally, *evolutionary allometry* relates to the covariation between shape and size across taxa. *Isometry* is a rigid transformation in which changes in proportions do not affect the shape. Understanding how changes in size at different levels have constrained morphological evolution is crucial to disentangle the underlying processes driving evolutionary changes.

*Developmental instability and asymmetries*

Some aspects of development, such as developmental instability (i.e., “the inability of an individual to produce a regular phenotype under given environmental conditions” [58]), can be assessed by examining asymmetries, deviations from bilateral symmetry. *Directional asymmetry* is the consistent appearance of an asymmetric component on one side. *Antisymmetry* is the appearance of an asymmetric component at equal frequencies between the right and left sides. *Fluctuating asymmetry*, more closely linked to developmental plasticity, represents a random variation of asymmetric components [59]. It also serves as a marker of stress, and can help predict individual fitness [60, 61].

*Heterochrony*

Heterochrony refers to changes in the rate, timing, or duration of developmental processes during ontogeny between an ancestral and a descendant species [62], or between related species or groups [63], ultimately influencing the course of evolution. There are two types of heterochrony: *paedomorphoses* and *peramorphoses*. Paedomorphoses refer to any processes such as neoteny, progenesis and postdisplacement in development that result in a more juvenile state of the descendant compared to the ancestor. The most famous example of paedomorphosis are axolotls, who retain juvenile features in the adult stage [64]. On the opposite, peramorphoses relate to accelerations, hypermorphoses and predisplacements to extension of the period of growth. However, studies of heterochrony in fossil specimens are not always straightforward, since ancestral relationships are often debated [65].

*Modularity and morphological integration*

Modules are combinations of traits defined by their common developmental or functional origins [66]. For example, regarding the craniofacial skeleton, three main modules are typically distinguished: the neurocranium, the cranial base or basicranium, and the viscerocranium (i.e., the face). Examining how traits covary, or integrate, within and between modules during ontogeny can provide insights into the constraints influencing morphological variation and the extent of plasticity. Highly integrated modules constrain morphological variation, often relating to functional needs (such as the upper and lower jaws). In the hominid cranium, a common pattern of craniofacial integration is observed across species [67, 68], which indicates a conservation of similar developmental patterns suggestive of developmental canalization, or stability [69, 70].

In contrast to Evo‐Devo, which represents a whole disciplinary field in evolutionary biology, auxology is a discipline of more restricted scope, but fundamental to addressing the limitations of the fossil record. Auxology, from the Greek Αὐξώ (*auxo*, “to grow”), is the scientific study of growth [74]. Growth studies began in the 18th century, with Stöller introducing the concept of “catch‐up growth” in 1729 [75], and Buffon conducting the first ever longitudinal study in 1777, shedding new light on ontogenetic processes such as puberty. The 19th century saw the rise of cross‐sectional studies as well as a focus on human growth's social and evolutionary aspects [75]. Growth research expanded significantly with the advent of anatomy laboratories and mathematical growth modeling in the late 19th century until the 1960s.

Auxology offers a valuable framework for paleoanthropologists studying hominin development. It focuses primarily on postnatal growth, the main aspect of development that paleoanthropologists are investigating. Additionally, auxology provides the empirical evidence that paleoanthropology often lacks, including “a robust database across multiple human populations, with cross‐sectional data and longitudinal data, with soft and hard tissue measures, and with individuals of known ages” [17]. In the early 2000s, the field of “paleoauxology” emerged, applying auxological methods to tackle the challenges of studying growth in ancient hominins [76]. The paleoauxology framework involves a theoretical and methodological shift in how morphological differences between populations are understood, from adult morphologies to ontogenetic processes that shape them. This approach emphasizes the study of similarities and differences between the morphologies of subadults across hominin taxa. This framework helps address key challenges in paleoanthropology, particularly the gap between modern and archeological samples. For instance, the anthropometric study of a skeletal modern sample suggests that studying growth in a skeletal population of non‐survivors may misrepresent the growth and health of survivors in that population [77]. By identifying such biases in the study of past populations, auxology plays a crucial role in overcoming the limitations of the fossil record.

### 3.2 Extracting New Information From Fossil Specimens: In Search of Developmental Markers

The use of diverse methodological frameworks aims to enhance the extraction of ontogenetic information from fossil remains. Advances in data extraction techniques, in turn, enable a more comprehensive application of these frameworks. For instance, the identification of new developmental markers in dental and bone tissues, which are correlated with key life history traits, allows for more accurate and reliable use of the life history framework.

Life history research, stemming from mathematical population studies and evolutionary ecology, investigates how natural selection has shaped the timing of life events [78]. This research focuses on traits such as size at birth and maturity, pace of growth, age at first and last reproduction, gestation length, number of offspring, interbirth interval, weaning, and life span. These traits reflect how energy is allocated to development, fitness and survival, and have evolved as trade‐offs, where investment in one trait may come at the expense of another. Compared to other primates, humans possess distinct biological traits, including an extended period of growth during childhood, a long life span, and a large brain, which have been extensively studied in paleoanthropology and primate life history in general [33].

However, life history events cannot be directly observed in fossils. Instead, “first‐order life history variables” must be inferred from “life history related variables” [3], such as body mass, brain mass, and dental development, including dental crown and root formation times, as well as dental eruption times. As previously discussed in Section 2.1, evaluating age‐at‐death for fossil individuals is a crucial step for reconstructing life history milestones. Analyzing dental eruption and maturation patterns can provide insight into an individual's age, as each tooth initiates at a specific age and in a specific order within the dentition [45].

However, evaluating the chronological age of fossil specimens using modern standards for crown and root calcification is challenging, as these parameters vary significantly among human populations due to differences in population history, geography, and chronology [79, 80]. This variation introduces potential bias in the analyzes of fossil remains. As a result, it was long thought impossible to accurately assess the timing of dental development in fossil specimens, and by extension, hominin life history events [47]. To address these limitations, the analysis of microscopic features in the enamel and dentine of tooth germs has significantly advanced paleoanthropological research, allowing scientists to access previously unattainable information and broaden the scope of life history traits studied in fossils. It is now possible to discuss whether a child was actually born or not, by detecting an accentuated line known as the “neonatal line” [81]. Moreover, this line can be used to calculate chronological ages by counting the numbers of incremental lines that individuals develop daily throughout their life [18]. Initially, this method relied on classical histology and tooth thin sections [82], which are destructive and not always allowed on fossil remains. However, since the 2010s, high‐resolution synchrotron imaging has granted non‐destructive access to tooth microstructure [83] (see Besnard et al. 2023 for a review [84]). The possibility to determine chronological ages for fossil individuals, and therefore to shed light on major life history traits—such as weaning (see Box 4), age at first reproduction, inter‐birth intervals, and number of offspring [94]—exemplifies how the identification of new developmental markers enables more reliable use of the life history framework.

Box 4 **Weaning**

Among the pivotal life history traits examined in human evolution, weaning marks the transition from dependence on breast milk to reliance on non‐milk foods. Across cultures and time periods, the specifics of weaning—such as its timing, gradualness, and introduction of solid foods—can vary [85]. Changes in breastfeeding and weaning practices over time can significantly impact child survival rates, morbidity, mortality, and even adult health [86].

Modern humans exhibit an early weaning pattern, coupled with prolonged dependence on care provided by the mother or other members of the community [87, 88]. This pattern, interpreted as being derived in modern humans, is thought to have emerged ~2.6 Ma with the genus *Homo*. It has likely evolved alongside increased meat consumption, scavenging behavior, and the development of stone tools [87].

Early weaning poses survival risks for offspring. Yet it was proposed that selection acted on the prolonged early brain growth that occurs in the very first years of life, necessitating nutrients beyond maternal milk [87]. Brain, craniofacial bones and meninges are integrated and develop in a tightly coordinated manner in terms of size and shape in the perinatal period [89]. Notably, modern human infants’ brains develop a globular shape within the first year of life, reflecting the rapid perinatal expansion of the parietal and occipital regions which are associated later in life with higher cognitive functions such as problem‐solving, social interaction and language [90]. This globular shape of the brain is a feature absent in Neanderthals and great apes [91]. Yet, this evolution in shape of a metabolically expensive brain involves additional energetic requirement to what breast milk alone can provide [88]. Thus, determining weaning timing in archeological and paleontological contexts is crucial for understanding life histories, social organizations, and adaptive capabilities, including resource management [86, 92].

Determining the age of weaning in past hominins and in human populations presents methodological challenges, particularly with archeological and fossil specimens. However, biomolecular analyzes use micro‐sampling strategies to track tooth growth increments and assess isotopic ratios (especially carbon and nitrogen), offering insights into dietary shifts towards adult foods [93].

Teeth offer more reliable information on life history traits than bones, as they preserve better in archeological contexts, and record chronological events, such as birth, without being altered by remodeling. However, some developmental markers can also be detected in bones. In the case of perinates or very young infants, age‐at‐death is most often estimated based on long bone length [95], as tooth buds are easily lost and may not be recovered during excavation [79]. In older individuals, epiphyseal fusion and skull synchondroses closure mark the achievement of skeletal maturity [48]. Similar to dental studies, the microscopic analysis of skeletal inner structures can indicate the approximate age class of an individual. Immature bone is characterized by the presence of woven or primary bone, a type of bone that is not completely mineralized [96]. Its presence can also indicate more rapid growth rates, as it is rapidly deposited to form new bone matrix [96]. Other microscopic features, such as osteocytes’ lacunae number [97], and the presence of secondary osteons [98], can also help discuss the approximate age of an individual [99].

### 3.3 Compensating for Missing Data: The Resources of Virtual Anthropology

Dealing with the limitations of the fossil record involves compensating for missing data by using computer‐based techniques to increase both the size and quality of available samples, along with applying specialized statistical analyzes. Techniques developed in virtual anthropology allow to address biases related to taphonomic alterations by conducting virtual reconstructions of fragmentary or deformed anatomical parts, improving the use of partial material [100, 101]. Basic principles involving mirror‐imaging (using symmetry between right and left countra‐lateral body parts) and warping (twisting and distorting a deformed object in an attempt to recover its original shape) are possible via advanced statistical analyzes [101]. Furthermore, increasing data on intraspecific variation across modern taxa (see Section 3.4.) contributes to robust frameworks for computing ontogenetic trajectories: fossil remains can be compared with well‐documented modern taxa, for which all developmental stages are available [89, 91, 102]. For example, advances in virtual anthropology allowed for the study of brain growth and shape changes from birth to adulthood, even based on fragmentary fossils [89], suggesting an extended period of growth much earlier in early hominin development than previously thought, as demonstrated in the Dikika child [102].

Several statistical analyzes of rare and fragmentary fossils are used to compensate for the lack of data. The first steps often involve estimations, such as for body size and mass, based on modern cohorts using linear regression [103]. Using data collected with virtual anthropology approaches, developmental simulations can be performed to grow a subadult along the trajectory of a chosen species [91]. To study variation using small sample sizes, and when missing data have been handled, principal component analysis (PCA) is the most commonly used exploratory tool in paleoanthropology [104, 105], as it allows for the projection of an individual into the known variance of another group/species. Other common statistical approaches such as resampling (randomization, permutation, bootstrapping) help to compensate for the small sample sizes.

### 3.4 Producing New Data: Cohorts and Experiments

To demonstrate direct interactions between geometric and developmental variations with environmental or behavioral factors, studies must be conducted on extant species. These can involve retrospective cohort studies or prospective experiments, both of which can help paleoanthropologists identify morphological variations in juvenile fossil remains and make inferences about their environment and behavior.

Studying large prospective cohorts within modern populations allows for the correlation of specific phenotypes with specific genotypes and factors such as environment, life habits, and health status. Regarding modern‐day children, several large international longitudinal cohorts are available (e.g., the Elfe cohort, “French Longitudinal Study of Children”, https://www.elfe‐france.fr/en/), most of them compiling information on their development, as early as infancy or even during the fetal period, and connecting this with their familial, social and economic environment and history. Such data are crucial for interpreting fossil remains, particularly in early ontogenetic stages. Indeed, these cohorts provide information on growth processes and growth patterns and their variations depending on specific known factors. For instance, this cohort was helpful in providing growth charts of the head circumferences of modern children from birth to the age of 5 years and assessing the influence of the maternal diet during pregnancy on the neurodevelopment of their child [106, 107].

Another possible approach is to lead experiments to actively control the modulation of a specific parameter and precisely evaluate its influence on bone or tooth developmental patterns. With these experimental studies, we can move beyond correlational observations to establish causal relationships. However, leading such experiments on pediatric patients is complicated, if not impossible, for ethical and practical reasons—particularly when modifications to routine medical care are involved. For instance, the use of sedation may be necessary to limit children's movements or reduce anxiety during the acquisition of measurements or images (X‐ray, MRI, CT‐scans).

As an alternative, animal models present an opportunity to perform longitudinal studies, sometimes spanning multiple generations, with control over crucial parameters such as genetic factors (e.g. study of inbred series [108]) and environmental conditions (e.g., temperature, diet, stress in reaction to captivity [109]). However, great attention must be paid to the choice of the animal model to study these growth and development processes. For instance, there are differences between avian and mammalian models, such as the presence or absence of a secondary ossification center in embryonic bone, or the irregular versus constant growth plate thickness [110]. Several parameters, including bone mass, architecture and composition, have to be taken into consideration when identifying the most appropriate animal model. Notably, only pigs and dogs exhibit bone density values closest to those of present‐day humans [111]. Nonetheless, thanks to these experiments, some studies have highlighted the significant roles of both genetic and environmental factors on biology, behavior and health. As an example, multi‐generational studies highlighted significant size and shape changes in the mandible and cranium of mice depending on diet hardness, with long‐term effects [108, 112]. This kind of research is crucial for understanding the adaptive mechanisms that may have shaped hominin craniofacial evolution, in particular in relation with dietary shifts maybe linked to environmental changes.

Computer‐based simulation offers another alternative to animal experimental models, allowing testing across various material parameters, mechanical loadings, etc. on *in silico* models. Among the various computer‐based approaches existing, Multibody Models represent the different body segments with non‐deformable elements (e.g., ellipsoids, cylinders) interconnected by virtual joints, to understand the overall behavior and kinematics of the body under specific loading conditions. For instance, a multibody dynamics analysis tested the efficiency of three different reconstructions of the iliacus and psoas muscles and their influence on the hip flexor musculature, which played a crucial role in the evolution of hominin bipedal gait and endurance running [113]. Finite Elements Models aim to understand the local behavior (i.e., in a very restricted physical area, not at the scale of a whole organ or tissue) of a specific biological organ (i.e., an isolated bone or tooth or a complex of several anatomical elements [114]). These computer‐driven simulations still depend on experimental data to define these material properties and validate the model. However, they can be valuable for testing hypothetical relationships between morphology and mechanical loading. As an example, this kind of model was used to test the hypothesis that hominin occlusal morphology may provide insight into their ability to crack large, hard food items [115].

## 4 Contemporary Debates on Development and Their Implications for Fossil Interpretation

### 4.1 Opportunities from the Extended Evolutionary Synthesis

In the preceding sections, we explored how paleoanthropologists address the limitations of the fossil record to deepen our understanding of hominin development. We argue that such limitations should not be viewed merely as obstacles to integrating biological information on development into paleoanthropology. In the final part of this paper, we discuss how contemporary understanding of the role of developmental processes in evolution may influence interpretations of the origins and significance of phenotypic variation among hominins.

Advances in developmental biology have led a number of scholars to call for an update of the theoretical framework built with the Modern Synthesis, by proposing an “Extended Evolutionary Synthesis” (EES) [116, 117]. A detailed analysis of the assumptions inherent in these two models is beyond the scope of this article, and we therefore only mention the aspects that are most relevant to the evolution and development of hominins. “Standard evolutionary theory” primarily emphasizes natural selection as the key mechanism explaining “why the properties of organisms match the properties of their environments (adaptation)” [117]. According to the EES, “developmental processes […] share with natural selection some responsibility for the direction and rate of evolution and contribute to organism–environment complementarity” [117]. Key processes likely to impact development include developmental bias (a limitation of variation in phenotypes that could not only constrain but also influence and facilitate evolution) [118], developmental plasticity (the capacity of an organism to alter its phenotype in response to environmental changes) [119], inclusive inheritance (a broader view of heredity encompassing cultural and epigenetic mechanisms alongside DNA transmission) [120], and niche construction (the process by which organisms and their environments co‐evolve) [121]. Traditionally, development has been viewed as mainly directed by a genetic ‘program’ driving phenotype construction. From this perspective, evolutionary relevant phenotypic novelty arises primarily from genetic mutations that modify elements of this program. In contrast, in the EES, the genome interacts dynamically with other factors—such as environmental influences, cellular processes, and organismal behavior—to shape development. In this view, the genome provides potentialities rather than strict instructions, meaning developmental outcomes emerge from the interplay between genetic and non–genetic factors.

Already widely discussed in biology and philosophy of biology [122], the EES made a breakthrough in the field of human evolution [31, 123, 124], evidenced by dedicated special issues in journals such as *Evolutionary Anthropology* [125] and *PaleoAnthropology* [126]. Contributing papers explore how key concepts from the EES—such as niche construction, developmental/phenotypic plasticity, and inclusive inheritance—offer new insights into hominin evolution and support alternative interpretations of the paleoanthropological record.

The relevance of the EES to the field of human evolution lies primarily in addressing the shortcomings of the Modern Synthesis—the theoretical framework that has underpinned paleoanthropology since the mid‐20th century [127], but that is now argued to restrict interpretations of the hominin fossil record in several aspects [124]. Firstly, the Modern Synthesis‐based interpretative framework centers on the role of natural selection in hominin evolution, assuming that any feature that has an evolutionary significance in the fossil record must have undergone selection. Consequently, selective pressures become the initial focus for explaining features in the fossil record. Secondly, this framework also assumes that any morphological variation in the fossil record is adaptive, results from natural selection and has a genetic basis [128]. Consequently, “in the pursuit of uncovering crucial adaptations, the implicit assumption has often been that [morphological] traits are fixed products of the genome” [29]. In many respects, paleoanthropology aligns with the “traditional interpretation” in evolutionary biology, which contends that the developmental generation of variations needs explanation, but cannot be used to explain diversity in morphology and adaptation [117].

In contrast, the EES emphasizes the role of developmental processes in the genotype‐to‐phenotype relationship and provides a broader theoretical framework for understanding the emergence of phenotypic traits observed in the fossil record. This approach reframes the conventional perspective in paleoanthropology: constrained by the fossil record to developmental snapshots that reflect the outcomes of developmental processes, paleoanthropologists have traditionally focused on the evolution of development itself—that is, on the distinct ontogenetic patterns in hominin taxa. The EES, however, shifts attention to the role that developmental processes potentially play in the evolution of the phenotypic traits we observe in the fossil record.

### 4.2 Expanding the Theoretical Framework Despite Fossil Record Limitations

So far, attempts to integrate the EES into the field of human evolution have mainly focused on behavioral, cultural and cognitive aspects. Much emphasis has been placed on gene‐culture co‐evolution, and on the concept of niche construction—i.e. the ability of organisms to modify their environment, impacting their own and other species’ evolution. In this context, *H. sapiens* is depicted as “an incredibly effective niche‐constructing species” [126].

In the effort to integrate the EES into the study of human evolution, less attention has been dedicated to the physiological and skeletal aspects of development. However, a promising avenue of research lies in exploring the role of phenotypic and developmental plasticity in human evolution. Phenotypic plasticity refers to the “ability of an organism to react to an internal or external environment input with a change in form, state, movement, or rate of activity” [119]. More specifically, developmental plasticity refers to the “subset of plastic phenotypic responses that involve irreversible modifications to growth and development” [123]. Plasticity leads to the production of distinct phenotypes in response to environmental conditions, for the same genotype (i.e. variations in adult anatomy that are not strictly genetically determined). However, this plasticity involves changes in gene expression, and downstream cell and tissue processes, as well as in the morphological, physiological, and behavioral traits that are products, in part, of gene expression. For instance, reaction norms for plasticity (describing the phenotypes that a genotype can produce across a range of environments) are based on underlying genetic architecture [10]. By generating phenotypic variation, behaviorally or environmentally driven plasticity may “lead the way” for gradual adaptation through natural selection and genetic change [123].

It could be argued that this theoretical framework has limited applicability to the study of human evolution due to the constraints of the fossil record and to the lack of experimental data in human developmental genetics. Assessing morphological variation in fossils is particularly challenging for reasons discussed in Section 2, [Link]. Additionally, the genotype‐phenotype relationship and the molecular mechanisms underlying phenotypic variation in hominins remain poorly understood. Together, these limitations complicate the effective application of insights from the EES. However, acknowledging the fossil record's limitations, as discussed above, is essential when evaluating how the EES might influence interpretations of hominin morphology. This perspective helps clarify which interpretive restrictions arise from paleontological constraints (explained just below)—and are therefore potentially intractable—and which stem from theoretical approaches and may be open to debate.

For instance, the small sample size (e.g. a single fossil supposedly representing a species) is a significant limitation when studying hominin development from fossil remains. This restriction often goes along with an interpretive bias, consisting in using an individual phenotype as a reliable genetic proxy for the species [31]. While integrating the EES into the paleoanthropological framework does not resolve the issue of limited samples, it encourages consideration of the full range of processes that may shape individual phenotypes. This broader perspective suggests more cautious interpretations of what a fossil phenotype may potentially reflect.

Bone functional adaptation is another example of how engaging with the EES challenges theoretical assumptions that shape our interpretation of the fossil record. Typically, bone remodeling is examined through a dichotomy that separates the functional and the developmental signal in bone morphology. Studies of bone functional adaptation focus on reconstructing past behaviors by examining functional signals in bone microarchitecture [26], while also including developmental patterns [27]. This dichotomous approach between “function” (a functional signal reflects phenotypic variations occurring during an individual's lifetime) and “development” (a developmental signal is seen as predetermined by the genotype) warrants reconsideration. Expanding the concept of development to include dynamic interactions with the environment allows us to reinterpret functional remodeling as a potential component and driver of bone developmental plasticity [29, 128]. When bones are shaped by interactions with their environment over short‐term timescales, these variations involve changes in gene expression and may have long‐term evolutionary implications by impacting selection on the resulting features. Thus, functional adaptation may, in some contexts, be better understood as developmental plasticity contributing to adaptive change [25]. This perspective highlights that studying development in human evolution requires both questioning existing conceptual frameworks (e.g., recognizing functional signals as part of developmental processes) and refining methods for data collection and analysis.

## 5 Conclusions

Here we explore the unique approach paleoanthropologists take to study development, shaped by the constraints of fossil evidence. Although these limitations prevent paleoanthropologists from contributing directly to developmental biology, which focuses on non–human embryonic development in experimental settings, paleoanthropology brings its own valuable perspective specific to the fossil record and evolutionary biology. By focusing on perinatal and postnatal growth stages, paleoanthropology also sheds light on how development may continue into adulthood and be influenced by environmental factors. While challenges such as the incomplete fossil record and the inability to experiment on fossil hominins make it difficult to establish precise genotype‐phenotype relationships, paleoanthropology is characterized by extensive efforts to extract any available information on hominin development. In this way, the field serves as a productive arena for exploring and refining hypotheses about development.

We believe that documenting the many limitations faced by paleoanthropologists, especially when studying development, is essential. Recognizing these constraints helps paleoanthropologists not only discriminate among the questions that can realistically be addressed using the fossil record, but also propose and explore new methodologies to circumvent these difficulties. As we have shown in this paper, efforts to overcome these challenges—through identifying new markers and supplementing incomplete or missing data—have been continuously driving progress in developmental studies within paleoanthropology.

Our approach raises several future challenges and perspectives, helping to link the distinctive aspects of development in paleoanthropology with broader developmental issues in biology and medicine. Engaging with new theoretical frameworks, such as the EES, aids in challenging default assumptions in interpreting phenotypic variation. Rather, it encourages consideration of a broader range of developmental processes that may have influenced some of the phenotypic outcomes observed in the fossil record. This shift calls for further research to operationalize the EES framework, such as by identifying which traits in hominin anatomy are particularly susceptible to plasticity, and exploring how these traits might help test alternative explanations for the evolution of hominin morphology.

Highlighting the complementarity between developmental biology and paleoanthropology is a promising research perspective. Viewing human evolution through the lens of developmental biology involves considering various interacting levels—such as both micro‐ and macroscopic factors—and aims for a more comprehensive understanding of ontogeny. This perspective treats the skeleton as part of a broader system, encompassing other organs (such as muscles and the brain) and physiological processes (including hormonal regulation) within their natural environment.

Conversely, viewing developmental biology through the lens of paleoanthropology requires understanding the organism within its evolutionary context. The study of human evolution offers a long‐term perspective that is essential for interpreting the biological significance of developmental patterns that are specific to present‐day humans, including prolonged childhood growth and extended life spans. Additionally, the evolutionary perspective is crucial for examining variations in human growth and for understanding diseases that arise from inadequate adaptation to new environments. The concept of ‘mismatch diseases’ or the ‘evolutionary mismatch hypothesis’ is based on the idea that modern humans grow in environments that differ drastically from those experienced by our ancestors [129].

To conclude, this review has highlighted that despite the limitations inherent to the study of the fossil record, paleoanthropology may not only benefit from, but also potentially contribute to developmental biology.

## Conflicts of Interest

The authors declare no conflicts of interest.

## Data Availability

The authors have nothing to report.
